# The Medical Year

**Published:** 1902-01-04

**Authors:** 


### Hospital Clinics and Medical Progress.

### THE MEDICAL YEAR.

It cannot be said that the past year has been distinguished by any great medical discovery, or that it is likely to stand out in history as marked off from its predecessors as the starting-point of any new departure either in medicine or surgery. Yet it has been a year of hard work and strenuous effort, and in hardly a single department do we now stand where we did a year ago. The growing interest taken by the public at large in medical and sanitary matters was well shown by the popularity attained by the British Congress of Tuberculosis. At this Congress one of the most striking events of the medical year took place in the unexpected announcement by Professor Koch of his doubts as to the transmissibility of bovine tuberculosis to man. Many of us, and, indeed, most of those who had devoted themselves to the public health aspects of medical science, had come to regard tuberculosis as one single disease, whether it occurred in cattle or in men—a belief which was on the verge of leading to some very serious action and to legislation which might have cost an enormous amount of money—when Professor Koch suddenly threw doubt upon the whole affair, questioned whether human and bovine tuberculosis were the same, denied that the disease as it occurred in cattle could be communicated to man, and informed his audience that he had advised the German Government not to take the steps which had been proposed with the object of guarding against what he looked on as a hypothetical danger. Professor Koch may be right or he may be wrong, but, at any rate, his speech has done one good thing, namely, has led to a reinvestigation of the whole subject by observers in all parts of the world. In this country a Royal Commission has been appointed, and until ts decision has been given all legislation on the subject will, of course, be held in abeyance—a small mercy for which we may imagine the Government will be duly thankful, for what with the butchers and the doctors, the consumers and the tax-payers, all pulling in opposite directions, tuberculosis is likely to prove a thorny topic for the law-maker. Next to tuberculosis the most interesting medical topic of the year has been typhoid fever. On the one hand the reports of many observers, especially those who have worked among our soldiers in the field, have gone to show that, true as may be the theory of water infection in ordinary circumstances, we must look in times of war to other modes of distribution, and more especially to dust and dirt and flies. On the other hand, the water-carriage theory has had a doughty champion in Dr. Leigh Canney, who has dealt with the matter with a thoroughness which deserves all praise, and has formulated a definite scheme for the protection of our armies from this scourge of typhoid fever; a scheme, be it said, which is the logical outcome of the theory on which it is based. How far this plan is practicable in the face of the exigencies of military life is a question which awaits solution, but one can hardly doubt that the methodical and exclusive use of recently-boiled water, as advocated by Dr. Canney, would eliminate the most universally recognised cause of typhoid fever epidemics. Whether in a country subject to such dust-storms as occur in South Africa, and where typhoid infection is so constantly present as seems to be the case there, the purification of the water would in itself be sufficient to prevent the spread of the disease is a point which trial alone can decide, and we are not without hope that after Dr. Canney's forcible remarks this subject also will be investigated afresh and authoritatively decided. Another medical question which is undergoing investigation at the hands of a Royal Commission is that of arsenical poisoning. It arose in regard to the Manchester epidemic of arsenical neuritis, but in view of the apparent ubiquity of arsenic and the many opportunities which exist for its introduction into the various manufactured compounds which man now delights to use as food it is probable that the future reports of the Commission will cover a much wider range than the one already presented, which refers to beer alone. In public health matters the events of the year have served to emphasise our lack of knowledge on certain points rather than to indicate that we possess any new powers over disease. Plague still remains a curse to some countries and a menace to the rest of the world. We know a good deal about plague, we recognise its clinical varieties, we can demonstrate its microbe, and we see that its epidemics run parallel with a prevalence of the disease among rats. But when we ask why the disease during the past seven years or so has invaded entirely new territories and held them so terribly in its grip as it has done, we have to confess that we do not know. The disease is obviously the same. Its behaviour is obviously different. Some new factors must have been introduced, probably connected with trade routes, and methods of commerce, and the hastening of communication between different parts of the world, but what the new factor is we cannot tell. So also in regard to small-pox. In the course of the last few months we seem to have run into the midst of a rising epidemic of this disease, and although the Metropolitan Asylums Board is working bravely and doing good work in controlling its progress, the events of this outbreak tend to demonstrate our lack rather than our surfeit of knowledge as to its epidemiology. It is easy to say that owing to the neglect of vaccination an epidemic was sure to arise. But if we ask why it arose at this particular time, in other words why the imported disease has on this occasion acted so differently and proved so much more difficult of control than it did the year before or any other year—for there are always plenty of cases to start an epidemic—we get no answer. It certainly cannot be maintained that the proportion of vaccinated individuals is so different now from what it was, say, eight years ago as to explain the wide diffusion of small-pox which has taken place during recent months, originating apparently from one or two cases which occurred last June, seeing that in the years between 1893 and 1899 the susceptibility of the population was apparently so slight that the numbers went steadily down from 2,815 in the first of those years to only 29 in the last. What then is the missing factor? That vaccination stands first in the management of small-pox we all maintain, but in the etiology of an epidemic there is evidently some other influence at work besides the presence of a badly vaccinated population. So far then as epidemiology is concerned the events of the past year do but illustrate how much there is yet to learn.

Surgical progress has centred largely in the abdomen. The year before we had had a perfect epidemic of phenomenal recoveries from bullet wounds, and it was a great shock and disappointment to the civilised world to find that in the case of President McKinley the assassin had succeeded in his foul attack. It is to be feared, however, that it must always be the case that even the most scientific and technically accurate surgery must prove helpless in the face of a constitutional feebleness of reparative power. In the operative treatment of diseases of the stomach many advances have been made, and much has been done to formulate the proceedings which are necessary in dealing by operation with some of the diseases of that organ. Especially has it been insisted upon by Mr. Mayo Robson that gastric hæmorrhage is a far more serious and fatal accident than some have imagined it to be, and that operations may very properly be had recourse to when the hæmorrhage recurs. An interesting if somewhat excited discussion has taken place in regard to the treatment of enlarged prostate by suprapubic prostatectomy, Mr. Freyer maintaining that what has to be done is to take out all the prostate, which is a new thing; while others hold that it is enough to take out a large portion of the prostate, which is a thing that has been done scores of times before. Hence a war of words which is to be regretted. Midwifery is now a branch of surgery, and probably the chief advance of the year in this department is the growing recognition of Cæsarean section, not merely as a last resource, but as a mode of treatment for cases in which the choice has hitherto been thought to lie between early induction of labour and the performance of craniotomy. Take it altogether we may say that while the past year has seen no great new discovery, it has been a year of work and progress, and that in almost every department of medical knowledge some advance has been made.

